# A Clinical Framework for Evaluating Cannabis Product Quality and Safety

**DOI:** 10.1089/can.2021.0137

**Published:** 2023-05-30

**Authors:** Caroline A. MacCallum, Lindsay A. Lo, Carly A. Pistawka, Michael Boivin

**Affiliations:** ^1^ Department of Medicine, Faculty of Medicine, University of British Columbia, Vancouver, British Columbia, Canada.; ^2^ Faculty of Pharmaceutical Sciences, University of British Columbia, Vancouver, British Columbia, Canada.; ^3^ Department of Public Health Sciences, University of Toronto, Toronto, Ontario, Canada.; ^4^ Faculty of Science, University of British Columbia, Vancouver, British Columbia, Canada.; ^5^ CommPharm Consulting, Barrie, Ontario, Canada.

**Keywords:** cannabis, THC, regulation, product safety, quality assurance, labeling

## Abstract

Increase in medical cannabis use, along with available products, warrants the need for clinicians to be knowledgeable in evaluating the quality of any cannabis product presented in clinical practice. Determining whether a product is regulated within the region is key in assessing overall quality and safety. Regulated products are held to a higher standard including independent testing, contamination mitigation, and concentration limits. Here, we present a clinical framework in evaluating cannabis products to ascertain the quality and regulation level of the product. Evaluation includes assessing the source company, reviewing product details (e.g., type, cannabinoid content, and labeling), and assessing quality control variables such as manufacturing and decontamination processes. The quality of products patients use is an important part of mitigating cannabis-related harms, especially in medically vulnerable patients. Currently, there is a great need to implement widespread standardization and regulations to ensure product quality and safety.

## Introduction

Cannabis use for medical purposes is increasing.^1–3^ As demand grows, so has the number of available cannabis products from both regulated and unregulated sources.^4–6^ This warrants the need for health care providers (HCPs) to properly evaluate the quality of any cannabis product presenting in clinical practice. Regardless of whether clinicians are recommending cannabis, or patients are self-medicating, they must be aware of the basic product safety considerations to mitigate potential harm.

With thousands of products on the market, the lack of product standardization has contributed to confusion surrounding what cannabis meets the highest quality standards for safety.^7^ Jargon and terminology used to describe cannabis products vary greatly depending on the region and regulations in place, adding to the confusion. Differences in packaging requirements between regulatory bodies have further complicated efforts to distill down clear markers of regulated and quality controlled products.

Societal narratives such as cannabis being “natural” or cannabidiol (CBD) being “safe,” compared with tetrahydrocannabinol (THC), may give both patients and HCPs a false sense of security.^7^ Many factors dictate overall patient safety. Quality control variables such as risk of contaminants or pesticides and product details such as dose or product type play an equally important role in determining risk for the individual patient.

An increasingly common clinical scenario involves patients bringing cannabis products to their health care provider (HCP) to ask questions. There is a paucity of information available to HCPs on how to properly evaluate whether or not the product meets the highest production standards. The following framework has been developed to allow HCPs to evaluate a product's quality. This piece does not discuss the efficacy of cannabis but instead focuses on finding the medical cannabis products with the strictest quality standards to mitigate risk. Although cannabis regulations are constantly changing around the world, this article primarily focuses on the established and federally regulated Canadian market when providing insights and examples.

### The importance of regulated products

Determining whether a product has been obtained from a regulated or unregulated source is a key factor in assessing its overall quality and safety. Assessment is complicated when cannabis is not regulated federally, as it is in Canada. For example, in the United States, some individual states have regulated cannabis, but it remains unregulated and illegal at a federal level. This has resulted in a lack of standardization. This makes the assessment of regulated products more difficult as they may differ per region. Regulations influence risk of contamination, product quality, and labeling accuracy.

An investigation on labeling accuracy in the United States uncovered that cannabinoid content (THC and CBD) was underlabeled in 25% and overlabeled in 60% of products tested (75 total).^8^ Overlabeling of products may lead patients to use products that will not provide them with the expected medical benefit. Alternatively, underlabeling poses a safety risk from unexpected impairment or adverse events. In regulated markets, such as Canada, products from regulated sources must uphold strong government-mandated regulations and pass standardized testing.^9,10^ These products must accurately display cannabinoid content and be free of or within the acceptable range for contaminants, pesticides, microorganisms, and diluents or fillers.^7,10,11^

Unfortunately, many cannabis products, especially from unregulated sources, lack standardized laboratory testing to ensure a final product meeting safety standards is produced. Companies have started to use terminology such as “sustainably grown” or “pesticide-free” on their products, but in many cases these terms do not reflect efficacy or purity due to lack of regulated testing.^7^ Without regulations and standardized testing, it is often up to the company's internal quality assurance and processes.

Chemical and microorganism contaminants are one of the primary concerns regarding unregulated products. Cannabis can be contaminated with microorganisms (pathogenic bacteria, yeasts, and molds) during any stage of production. Microorganism-contaminated products can be dangerous for medical patients, especially those with immunocompromising conditions or using immunosuppressive therapies, as they are at high risk of infection. Owing to the risk of microorganism contamination, decontamination processes using pesticides are not uncommon throughout the growth process.

This is worrisome in unregulated markets, where regulations and testing requirements may not be followed to ensure the type of pesticide and levels are within acceptable ranges.^10,11^ Pesticide-contaminated products are also of concern, especially for young patients with neurological conditions.^7^ Adherence to regulations limits the risk of contamination while also improving clarity and reliability within products.

### Proposed tool for product safety and quality evaluation

Product quality evaluation combines the assessment of quality alongside patient-specific considerations. The primary goal is to determine whether the cannabis product is from a regulated source with mandatory standardized laboratory testing. If a patient appears to be using an unregulated sourced product, it is strongly recommended to switch to a regulated product. The proposed steps to assessing when a cannabis product meets quality standards are outlined in Table 1.

**Table 1.** Cannabis Quality and Safety Framework

Q1: What type of product is it?
a. Are there any concerns with the specific product type?
Q2: Does the product have appropriate labeling?
b. Does it show the name of the product?
c. Does it show the name of the producer/distributor?
d. Is the company's contact information listed (website, phone, email)?
e. Does the product have health warning labels (e.g., THC logo)?
f. Are there any additional warnings?
g. Are optimal storage details listed?
Q3: What is the listed cannabinoid content?
h. If dried flower or inhaled concentrates, is THC and/or CBD listed (% or mg/g)?
i. If ingestible oils, is the mg/mL of THC and/or CBD listed?
j. If edibles, is there a “serving size” or “dose” listed?
k. If topicals/creams, is there a THC and/or CBD amount listed (mg, mg/mL, mg/g)?
Q4: What are the listed product/manufacturing details?
l. Is there a packaging date?
m. Is there an expiry date (including “no expiry date”)?
n. Is there a lot/batch number?
o. Is the net weight/volume listed?
p. If the product is an oil, edible, or vape, are the noncannabis ingredients listed?
q. Is the decontamination method specified (label or company website)?
r. Is there evidence of third party testing (label or company website)?
Q5: Is packaging in line with regional regulations?
s. Does packaging have a security feature to indicate whether the seal is broken?
t. Does the product have child-resistant packaging?
u. Does the packaging and labeling appeal to children/adolescents (cartoon images, vibrant colors, packaging similar to candy, etc.)?
v. Is the product labeled as being within the regional allowable THC limits?

CBD, cannabidiol; THC, tetrahydrocannabinol.

#### Q1. What type of product is it?

The first step in product evaluation is to assess the type of cannabis product. Product type can be useful in further affirming whether it is regulated and is important for assessing patient-specific safety risks. Common product types used for medical use are oral oil formulations or dried cannabis flower. HCPs should be aware of common cannabis concentrates (e.g., dabs, waxes, and shatters), which are considered higher risk products (Table 2) and are generally not recommended for medical cannabis use.

**Table 2.** Cannabis Concentrates

Cannabis concentrate	Description
Solvent-based extracts	
“Live” resin	Hydrocarbon extract. Extracted from fresh cannabis plant material. Preserves higher concentration of terpenes
Shatter 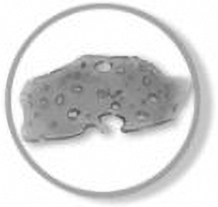	Hydrocarbon extract. A golden, translucent, brittle concentrate. Brittle due to crystallization of THCA in the extract Up to 90% potency
Wax 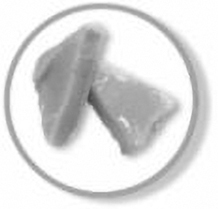 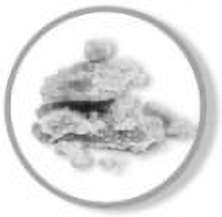	Hydrocarbon extract. Soft concentrate that varies in appearance, texture, and color depending on processing technique Common forms include: Budder: Whipped into a smooth consistency with a high terpene concentration. Also termed: *badder, frosting, icing,* and more Crumble: Purged wax (removal of any residual solvents) to create a drier texture concentrate. Have porous appearance like a honeycomb Both are generally 60–90% THC potency
Carbon dioxide (CO_2_) extraction (most common method of medical cannabis oil production)	Uses carbon dioxide under extreme temperature and pressure to extract cannabinoids and terpenes from the plant. Commonly used to create cannabis oils and vaporization products. Wide range of THC and CBD potency
Distillate 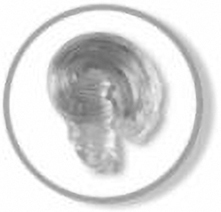	Viscous, translucent, and flavorless oil. Concentrates made through an extensive refinement process in which crude extracts such as CO_2_ and ethanol are distilled. Often used in edibles, topicals, and vaporization cartridges In general, 70–90% THC potency
Isolate 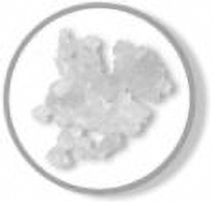	Commonly uses a vacuum pump that pulls cannabis through fine sand filtration. Cannabinoids can be isolated into concentrated crystalline structures or powder. Other cannabinoids and plant impurities are removed. Can add cannabis terpenes to the final product. Nearly 100% THC or CBD potency
Solventless extracts	
Bubble hash/ice water hash 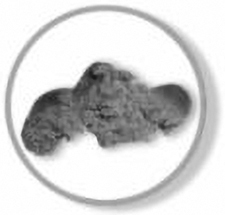	Created by agitating cannabis buds in ice water and filtering water through fine screen bags Water is then filtered and trichomes of the cannabis flower are collected leaving a paste known as “hash” In general, 40–60% THC potency
Kief/dry sift 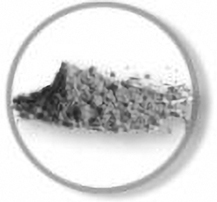	Kief: Flower is ground and sifted, leaving behind complete trichome glands Dry sift: Mechanically separated and collects resin glands from the cannabis flower using a series of different sized mesh screens. Only small trichome heads can pass through In general, 50–80% THC potency
Rosin 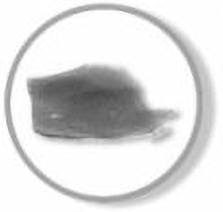	Extracted using high pressure and gentle heat to squeeze terpene-rich cannabinoid concentrate from cannabis flowers In general, 40–75% THC potency

© Caroline MacCallum, used with permission. Information gathered from Refs.^17,18^

These products are not recommended for medical cannabis use.

Concentrates are either solvent-based or solventless extracts. The extraction process may introduce harmful solvents and contaminants, such as naphtha, butane, and petroleum ether.^7^ At-risk populations such as those with significant comorbidities or the elderly may be at increased risk of harm from such contaminants. Furthermore, cannabis concentrates typically contain very high amounts of THC, especially in unregulated markets. Higher THC dose leads to increased risk of adverse effects and has many more safety considerations.^12,13^

Although vaping products are becoming increasingly common, unregulated versions of these products pose a significant health risk known as e-cigarette and vaping-associated lung illness (EVALI).^14,15^ The Centers for Disease Control and Prevention suggests that unregulated vaping products were associated with a higher risk of EVALI, which is likely a result of high vitamin E acetate levels.^16^

#### Q2. Does the product have appropriate labeling?

**FIG. 1.**
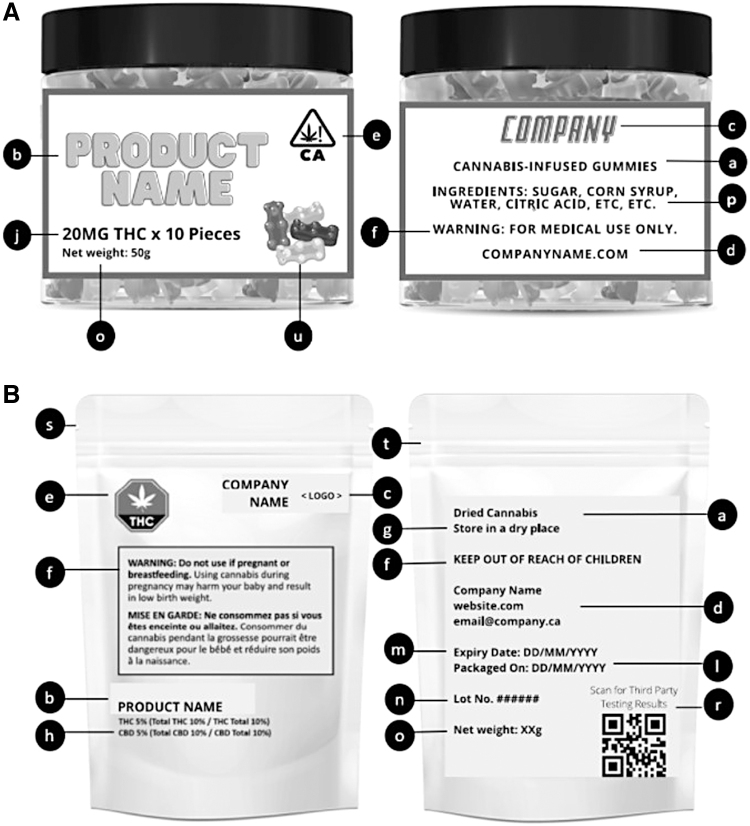
**(A)** Example of nonregulated cannabis product label **(B)** Example of regulated (Canada) cannabis product label. Lowercase letters refer to components in Table 1: a. Product type b. Name of product c. Name of grower/producer d. Company contact information e. Health warning logo f. Additional warning g. Storage suggestion h. THC/CBD content (flower) j. THC/CBD content (edible) l. Packaging date m. Expiry date n. Lot number o. Net weight p. Noncannabis ingredients (for oil, edible, vape) r. Third-party testing s. Security feature (seal) t. Child-resistant packaging u. Packaging with/without child appeal. CBD, cannabidiol; THC, tetrahydrocannabinol.

Regulated products should clearly list the product's name, the producer and/or distributor, and the company's contact information (See Fig. 1, nonregulated and regulated label examples, components b, c, d). Products that do not have this information are likely to be from unregulated sources. If possible, company website, regulatory status (i.e., licensed cannabis producer or retailer), and adherence to local regulatory and product testing guidelines should be verified. Indications of optimal storage requirements can help ensure preservation of product quality (See Fig. 1, nonregulated and regulated label examples, component g).

**FIG. 2.**
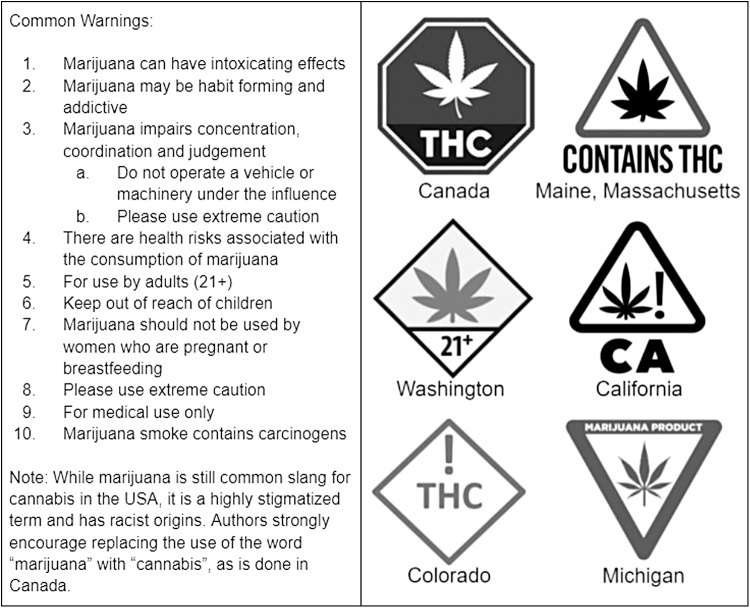
Common cannabis warning labels.

Other red flags to consider include media reports highlighting contaminants or inaccurate cannabinoid content for a particular producer and should be a cause for question. As with any health product, including cannabis, adequate health warning labels should be provided (See Fig. 1, nonregulated and regulated label examples, component e and Fig. 2, common cannabis warning labels). Although the presence of health warnings does not confirm whether a product is regulated, the absence of health warnings is a strong indicator the product is not regulated.

#### Q3. What is the listed cannabinoid content?

Cannabinoid content is crucial in determining the appropriateness of cannabis and its related risk. It is important to consider the listed THC and/or CBD amount, whether it be in percentage, concentration, or weight (See Fig. 1, nonregulated and regulated label examples, components h–k). Dried flower product content is commonly listed as percentage or mg/g of THC and CBD. This indicates the amount of CBD or THC in the overall weight of the product. Ingestible oils or tinctures are commonly listed as mg/mL, determined by the milligram of CBD or THC per milliliter.

Edible products are measured by the milligram of THC and CBD within one serving, but may provide this as total milligram of THC and CBD, and the number of servings in the package. In cases wherein CBD and THC contents are listed, such as “THC%:CBD%” or “CBD%:THC%,” it is important to review which percentage or concentration is associated with which cannabinoid, as not all products use the same labeling method (e.g., some list THC first whereas others list CBD first).

Regulated dried cannabis flower concentrations generally range from 0.01% to 30% THC and/or CBD,^19,20^ whereas regulated extracts, topicals, and oils generally have a maximum THC content of 1000 mg per container.^21^ In contrast, unregulated markets often have higher potency products.^20^ Allowable THC content may vary by region. HCPs should be aware of limits within their region as this can be helpful in spotting unregulated products.

Very high THC potency (e.g., >30% for dried flower) is not recommended for medical use due to its link with greater adverse effects and dependence.^9,22–24^ Patient-specific risks for using high THC products include those with unstable cardiac conditions, current or history of substance dependence, or concurrent unmanaged mental health conditions, especially psychosis or bipolar.^9,24,25^ Detailed information on cannabis-related precautions and contraindications as well as drug interactions can be found in MacCallum et al.^24^

#### Q4. What are the listed product/manufacturing details?

Detailed product specifications are helpful in ensuring product quality. Labeling requirements will vary greatly depending on region. In general, regulated products will have more detailed product labels than unregulated products. Countries with federally regulated labeling practices, such as Canada, serve as good examples to base assessments on. Labeling components include packaging date, expiry date, lot number, net weight or volume, noncannabinoid ingredients, decontamination method (e.g., gamma irradiation, pesticides), and verification of third party testing (See Fig. 1, nonregulated and regulated label examples, label components l–o, r).

Packaging and expiry dates are important for assessing when the product should no longer be used. Third party testing is a crucial step in quality control and product safety. Pesticides, microbes, and heavy metals should all be tested for. It is best practice for products to have a certificate of analysis (COA) from a third party laboratory showing the results obtained from quality control testing (See Supplementary Appendix SA1, COA from a third party laboratory). Some of this information may only be found on the company website or accessible through a QR code (i.e., decontamination method and COA).

Cannabis could be infected with microorganisms, therefore, decontamination processes (e.g., gamma irradiation, pesticides) are often used.^26^ Of particular concern is the use of pesticides, which has been found in both unregulated and regulated products.^27–29^ In 25 samples of cannabis products which were tested from regulated dispensaries in Washington, 22 (86%) were found to contain pesticides. Many of these exceeded the upper allowable limit.^27^ Testing showed that contaminants, such as pesticides, were much more commonly found in products from unregulated dispensaries than in products from regulated dispensaries.^10,11^ While dispensaries may be regulated at a state level, the lack of federal regulation in the United States causes difficulty in ensuring product quality.

Pesticide-free cannabis products are the best choice. Gamma radiation, a common method for food product sterilization, is the preferred decontamination process.^26,27,30,31^ This process uses ionizing radiation exposure to kill pathogens and maintain a low microbial contamination level.^7^ Available evidence supports that irradiated cannabis is safe and does not alter THC or CBD content.^30,31^ Some reductions in terpenes have been reported.^30^ However, the significance of this reduction remains to be determined and gamma radiation is still considered the best method of decontamination.^7,27^

In patients who are immunocompromised, care should be taken to ensure products being used are at the lowest risk of contamination. Clinicians should look for indication gamma radiation decontamination processes were used on either the label or company's website, which may be depicted by the gamma symbol (γ). Furthermore, confirmation of third party product testing is important to evaluate the absence of harmful contaminants and the accuracy of labeled cannabinoid content.

#### Q5: Is packaging in line with regional regulations?

Most regions that regulate cannabis have specifications for packaging considerations or security features. If the product is unopened, packaging should be inspected for security feature(s) to indicate it was sealed before purchase (See Fig. 1, nonregulated and regulated label examples, label component s). If the patient has already used the product, ask the patient whether they noted a security feature when they first opened it.

In addition to a security feature, regulated products often require child-resistant packaging to prevent children from consuming the product (See Fig. 1, nonregulated and regulated label examples, label component t). This is often accompanied by requirements for opaque packaging so that product cannot be seen. In addition, “desirable” images or logos (i.e., candy or cartoons) that could be appealing to minors are usually not permitted by regulators.

Being familiar with product and labeling regulations within one's region is essential for spotting unregulated products. Knowledge of federal regulations, such as in Canada, or regional regulations, such as in the United States, is helpful in assessing whether a product is from the regulated market. Labeling requirements in Canada, often considered the gold standard, can be found at “Packaging and labelling guide for cannabis products” by Health Canada.^32^ Labeling requirements by state can be found at “Cannabis Labelling Requirement by State” by Weber Packaging Solutions.^33^ Ensuring the product is within the regionally approved THC limit and adheres to all required labeling requirements is a good additional strategy to confirm whether the product is regulated.

## Conclusion

Increased cannabis product availability, combined with lack of standardization, has led to difficulties in determining whether a product meets adequate quality standards. The Proposed framework acts as a model for HCPs to use when assessing a cannabis product for a patient. By understanding regional or federal laws regarding cannabis, HCPs can differentiate regulated and unregulated products. Regulated products have greater quality control including independent testing, contamination mitigation, and concentration limits. Product labeling, product type, cannabinoid content, manufacturing processes, and region-specific product allowances should all be considered when evaluating patient-specific risks.

One limitation to the proposed framework is it was extrapolated from the Canadian market. Therefore, the insights provided may not be applicable to all countries. However, given the paucity of information available on this topic, using a well-established and federally regulated market that has been legalized since 2014 allowed for a more detailed discussion with clearer examples of key product considerations.

The quality of cannabis products is an important part of mitigating patient harm. The proposed framework has been translated into a free practical clinical tool to allow questionable products to be scored. This can be found at (safe-cannabis.com). There is a greater need now more than ever to implement widespread standardization and regulation for cannabis products. As demand increases and more countries head toward regulation, standardization will be a crucial component to ensuring product quality and safety.

## Supplementary Material

Supplemental data

## Supplementary Material

Supplementary Appendix SA1

## Abbreviations Used

CBD: cannabidiol
CDC: Centers for Disease Control and Prevention
COA: certificate of analysis
EVALI: e-cigarette and vaping-associated lung illness
HCP: health care provider
THC: tetrahydrocannabinol

## References

[1] Han BH, Palamar JJ. Trends in cannabis use among older adults in the United States, 2015–2018. JAMA Intern Med. 2020;180:609–611. 32091531 10.1001/jamainternmed.2019.7517 PMC7042817

[2] Lintzeris N, Mills L, Suraev A, et al. Medical cannabis use in the Australian community following introduction of legal access: the 2018–2019 Online Cross-Sectional Cannabis as Medicine Survey (CAMS-18). Harm Reduct J. 2020;17:37. 32513180 10.1186/s12954-020-00377-0 PMC7278204

[3] Health Canada. Government of Canada. Canada.ca. 2020. https://www.canada.ca/en/health-canada/services/drugs-medication/cannabis/research-data/canadian-cannabis-survey-2020-summary.html#a2 Accessed August 15, 2021.

[4] Hammond D. Communicating THC levels and ‘dose’ to consumers: implications for product labelling and packaging of cannabis products in regulated markets. Int J Drug Policy. 2021;91:102509. 31351756 10.1016/j.drugpo.2019.07.004

[5] Borodovsky JT, Lee DC, Crosier BS, et al. U.S. cannabis legalization and use of vaping and edible products among youth. Drug Alcohol Depend. 2017;177:299–306. 28662974 10.1016/j.drugalcdep.2017.02.017 PMC5534375

[6] Russell C, Rueda S, Room R, et al. Routes of administration for cannabis use - basic prevalence and related health outcomes: a scoping review and synthesis. Int J Drug Policy. 2018;52:87–96. 29277082 10.1016/j.drugpo.2017.11.008

[7] MacCallum CA, Lo LA, Betts F, et al. Chapter 31: Product safety and quality control. In: Narouze S, MacCallum C, eds. Cannabinoids and pain. Springer: Switzerland, 2021, pp. 249–258.

[8] Vandrey R, Raber JC, Raber ME, et al. Cannabinoid dose and label accuracy in edible medical cannabis products. JAMA. 2015;313:2491–2493. 26103034 10.1001/jama.2015.6613

[9] Health Canada. Information for health care professionals: cannabis (marihuana, marijuana) and the cannabinoids. 2019. https://www.canada.ca/content/dam/hc-sc/documents/services/drugs-medication/cannabis/information-medical-practitioners/information-health-care-professionals-cannabis-cannabinoids-eng.pdf Accessed July 28, 2021.

[10] Eykelbosh A. Unregulated cannabis: Risky production practices raise concern for consumers [blog]. National Collaborating Centre for Environmental Health: Vancouver, BC, 2021. https://ncceh.ca/content/blog/unregulated-cannabis-risky-production-practices-raise-concern-consumer Accessed July 28, 2021.

[11] Craven C, Birjandi A, Simons B, et al. Determination of eighty-two pesticides and application to screening pesticides in cannabis growing facilities. J Environ Sci. 2021;104:11–16. 10.1016/j.jes.2020.11.004 33985714

[12] Loflin M, Earleywine M. A new method of cannabis ingestion: the dangers of dabs? Addict Behav. 2014;39:1430–1433. 24930049 10.1016/j.addbeh.2014.05.013

[13] MacCallum CA, de Freitas L, Lo LA, et al. Chapter 36: cannabinoid-related adverse events and impairment. In: Narouze S, MacCallum C, eds. Cannabinoids and pain. Springer: Switzerland, 2021, pp. 293–306.

[14] Layden JE, Ghinai I, Pray I, et al. Pulmonary illness related to E-cigarette use in illinois and Wisconsin—preliminary report. N Engl J Med. 2020;382:903–916. 31491072 10.1056/NEJMoa1911614

[15] Blount BC, Karwowski MP, Shields PG, et al. Vitamin E acetate in bronchoalveolar-lavage fluid associated with EVALI. N Engl J Med. 2020;382:697–705. 31860793 10.1056/NEJMoa1916433 PMC7032996

[16] Centers for Disease Control and Prevention (CDC). Outbreak of lung injury associated with the use of E-cigarette, or vaping, products. CDC: GA, 2020.

[17] Craft S, Winstock A, Ferris J, et al. Characterising heterogeneity in the use of different cannabis products: latent class analysis with 55,000 people who use cannabis and associations with severity of cannabis dependence. Psychol Med. 2020;50:2364–2373. 31607281 10.1017/S0033291719002460

[18] Nickus L. What are cannabis concentrates and how do you consume them? Weedmaps. 2020. https://weedmaps.com/learn/products-and-how-to-consume/cannabis-concentrates Accessed February 12, 2021.

[19] Mahamad S, Wadsworth E, Rynard V, et al. Availability, retail price and potency of legal and illegal cannabis in Canada after recreational cannabis legalisation. Drug Alcohol Rev. 2020;39:337–346. 32291811 10.1111/dar.13069

[20] Smart R, Caulkins JP, Kilmer B, et al. Variation in cannabis potency and prices in a newly legal market: evidence from 30 million cannabis sales in Washington state. Addiction. 2017;112:2167–2177. 28556310 10.1111/add.13886 PMC5673542

[21] Health Canada. Final Regulations: edible cannabis, cannabis extracts, cannabis topicals. 2019. https://www.canada.ca/content/dam/hc-sc/documents/services/drugs-medication/cannabis/resources/final-regulations-edible-cannabis-extracts-topical-eng.pdf Accessed August 15, 2021.

[22] World Health Organization. WHO Expert Committee on Drug Dependence: fortieth report. Geneva, 2018.

[23] Fischer B, Russell C, Sabioni P, et al. Lower-risk cannabis use guidelines: a comprehensive update of evidence and recommendations. Am J Public Health. 2017;107:e1–e2. 10.2105/AJPH.2017.303818 PMC5508136 28644037

[24] MacCallum CA, Lo LA, Boivin M. “Is medical cannabis safe for my patients?” A practical review of cannabis safety considerations. Eur J Intern Med. 2021;89:10–18. 34083092 10.1016/j.ejim.2021.05.002

[25] National Academies of Sciences, Engineering, and Medicine (NASEM). The health effects of cannabis and cannabinoids: the current state of evidence and recommendations for research. The National Academies Press: Washington, DC, 2017. 10.17226/24625. 28182367

[26] Sarma ND, Waye A, ElSohly MA, et al. Cannabis inflorescence for medical purposes: USP considerations for quality attributes. J Nat Prod. 2020;83:1334–1351. 32281793 10.1021/acs.jnatprod.9b01200

[27] Russo EB. Current therapeutic cannabis controversies and clinical trial design issues. Front Pharmacol. 2016;7:309. 27683558 10.3389/fphar.2016.00309 PMC5022003

[28] Moulins JR, Blais M, Montsion K, et al. Multiresidue method of analysis of pesticides in medical cannabis. J AOAC Int. 2018;101:1948–1960. 29843862 10.5740/jaoacint.17-0495

[29] Craven CB, Birjandi AP, Simons B, et al. Determination of eighty-two pesticides and application to screening pesticides in cannabis growing facilities. J Environ Sci (China). 2021;104:11–16. 33985714 10.1016/j.jes.2020.11.004

[30] Hazekamp A. Evaluating the effects of gamma-irradiation for decontamination of medicinal cannabis. Front Pharmacol. 2016;7:108. 27199751 10.3389/fphar.2016.00108 PMC4847121

[31] Ruchlemer R, Amit-Kohn M, Raveh D, et al. Inhaled medicinal cannabis and the immunocompromised patient. Support Care Cancer. 2015;23:819–822. 25216851 10.1007/s00520-014-2429-3

[32] Health Canada. Packaging and labelling guide for cannabis products. 2019. https://www.canada.ca/en/health-canada/services/cannabis-regulations-licensed-producers/packaging-labelling-guide-cannabis-products/guide.html Accessed June 31, 2021.

[33] Weber Packing Solutions. Cannabis Labeling Requirements By State. Weber Packaging Solutions. 2018. www.weberpackaging.com/pdfs/Cannabis%20Laws%20by%20State.pdf Accessed May 24, 2021.

