# Supplementary material for: A Clinical Framework for Evaluating Cannabis Product Quality and Safety
Source: Cannabis Cannabinoid Res. 2023 May 30;8(3):567–74. doi: 10.1089/can.2021.0137 (PMC10249738; doi:10.1089/can.2021.0137)
Supplement: Supplemental data [file Suppl_AppSA1.docx]

Appendix 1. Certificate of Analysis (COA) from a Third Party Lab

| Certificate of Analysis  Client:  Product Name:  Sample ID: ############  Lot #: ##### Authorized By: _____________  Laboratory Manager, QA  CoA Prepared: DD-MM-YYYY  Potency wt % mg/mL  Total THC equivalents (D9-THC + D9-THCA x 0.877) 0.13% 1.0  Total CBD equivalents (CBD + CBDA x 0.877) 2.55% 24.1  Most abundant minor cannabinoids  wt % wt %  CBC 0.14% CBGA BLQ  CBG BLQ CBDV BLQ  Terpenes  Most abundant of the 39 terpenes quantified  wt % wt %  Guaiol 0.024 alpha-Humulene 0.004  alpha-Bisabolol 0.022 Borneol isomers 0.001  trans-Caryophyllene 0.010 beta-Myrcene BLQ  alpha-Terpineol 0.007 Fenchone isomers BLQ  Linalool 0.006 Fenchyl Alcohol BLQ  Contaminant Analysis  Residual Solvents  Limits for residual solvents below ICH Q3C guidelines pass  Microbial Quality  Total aerobic microbial counts pass  Total yeast and mold counts pass  Bile-tolerant gram-negative bacteria pass  E coli absent  Salmonella spp absent  Aflatoxins Aflatoxin B1, B2, G1, G2 pass    Heavy Metals Arsenic, Cadmium, Lead, Mercury pass  Pesticides None detected |
| --- |

| Details of Testing  Cannabinoid Profile  Quantification of 14 cannabinoids by ultra-high-performance liquid chromatography and mass spectrometry detection (UHPLC-MS). LOQ for flower and formulated oils is 0.064% (w/w) and for concentrates is 0.128% (w/w). [STM-401]  Terpene Profile  Quantification of 39 terpenes by gas chromatography and mass-spectrometry detection (GC-MS). [STM-406]  Residual Solvents  Quantification of 11 extraction solvents using headspace sampling, gas chromatography, and mass spectrometry detection (HS-GC-MS) compliant to ICH Q3C. [STM-410]  Microbial Quality  Microbiological screening using European Pharmacopoeia methods 2.6.12, 2.6.13, and 2.6.31. [STM-402]  Aflatoxins  Aflatoxins B1, B2, G1, and G2 quantification using immunoaffinity column chromatography followed by ultra-high- performance liquid chromatography with tandem mass-spectrometry (UHPLC-MS/MS) detection to meet criteria in European Pharmacopoeia method 2.8.18. [STM-405]  Heavy Metals  Microwave digestion and inductively-coupled plasma mass-spectrometry detection (ICP-MS) to test for arsenic, cadmium, lead, and total mercury. ICP-MS analysis performed by ISO 17025 accredited 3rd party lab. [STM]  Pesticides  Screening of 51 pesticide residues and plant growth regulators specifically identified as prevalent contaminants to cannabis production. Analysis performed ultra-high-performance liquid chromatography with tandem mass- spectrometry detection (UHPLC-MS/MS). [STM-407]  Pesticides and Plant Growth Regulators tested for:  Abamectin Diazinon Imidacloprid Pyrethrin I  Acephate Dichlorvos Kresoxim-methyl Pyrethrin II  Acetamiprid Dimethoate Malathion Pyridaben  Aldicarb Ethoprophos Metalaxyl Spinosad A  Azoxystrobin Etofenprox Methiocarb Spinosad D  Bifenazate Etoxazole Methomyl Spiromesifen  Boscalid Fenoxycarb Myclobutanil Spirotetramat  Carbaryl Fenpyroximate Oxamyl Spiroxamine  Carbofuran Fipronil Paclobutrazol Tebuconazole  Chlorantraniliprole Flonicamid Phosmet Thiacloprid  Chlorpyrifos (ethyl) Fludioxonil Piperonyl butoxide Thiamethoxam  Clofentezine Hexythiazox Propiconazole Trifloxystrobin |
| --- |
